# Visualizing genotype × phenotype relationships in the GAW15 simulated data

**DOI:** 10.1186/1753-6561-1-s1-s132

**Published:** 2007-12-18

**Authors:** Xuejun Qin, Silke Schmidt, Eden Martin, Elizabeth R Hauser

**Affiliations:** 1 Center for Human Genetics, Duke University Medical Center, Durham, NC 27710, USA

## Abstract

We have developed a graphical display tool called SIMLAPLOT for visualizing different ways in which continuous covariates may influence the genotype-specific risk for complex human diseases. The purpose of our study was to examine continuous covariates in the Genetic Analysis Workshop 15 simulated data set using our novel graphical display tool, with knowledge of the answers. The generated plots provide information about genetic models for the simulated continuous covariates and may help identify the single-nucleotide polymorphisms associated with the underlying quantitative trait loci.

## Background

One of the most challenging aspects of complex genetic traits is developing intuition regarding genotype × phenotype relationships across the distribution of a continuous covariate. Standard family-based and case-control association tests do not directly examine the role of continuous disease-related covariates in genetic models. Such covariates may themselves have a genetic basis in the form of a quantitative trait locus (QTL), or they may interact statistically with one or more susceptibility genes (gene × environment (G × E) interaction). A third possibility is that they may define more homogeneous subgroups of patients or families, in which the main effect of a particular susceptibility gene is more easily detected. While family-based designs offer protection against spurious associations as a result of population stratification, they are known to be less efficient than case-control designs for some disease models. Case-control designs may also have advantages over family-based designs in terms of distinguishing QTL models from G × E interaction models. To improve our understanding of a variety of complex genetic models used in simulation studies, we developed a novel graphical display tool, SIMLAPLOT, which produces plots of the relationship between affection status, continuous covariate values, and marker genotypes. SIMLAPLOT provides a way to examine genetic model parameters for continuous traits and to evaluate models by comparing plots from observed data to theoretical model plots. By applying SIMLAPLOT to the simulated Genetic Analysis Workshop 15 (GAW15) data, our goal was to identify the SNPs in highest linkage disequilibrium (LD) with simulated QTLs underlying measured continuous covariates and to characterize the corresponding genetic models qualitatively.

## Methods

SIMLAPLOT is a supplement to the simulation program SIMLA [1]. SIMLA uses a prospective logistic regression model as the penetrance function. This allows for the implementation of flexible multivariable genetic models, which may include terms derived from genotypes at a known susceptibility locus or a nearby marker, non-genetic covariate terms, and product terms modeling interaction between genotypes and covariates. The penetrance function can be expressed as follows:

$$P(AFF|G,E)=\frac{\exp(\beta_{0}+\beta_{1}G+\beta_{2}E+\beta_{3}G\times E)}{1+\exp(\beta_{0}+\beta_{1}G+\beta_{2}E+\beta_{3}G\times E)},$$

where *AFF *= 1 if affected and *AFF *= 0 otherwise. *G *codes for the three possible genotypes (dd, Dd, DD) at a bi-allelic susceptibility locus or nearby marker based on the user-specified mode of inheritance (additive, dominant, or recessive). *β*_1 _is the log-transformed odds ratio for the susceptibility locus. *E *is a continuous, normally distributed covariate; it can be an environmental risk factor, an endophenotype, or a quantitative trait, which depends on an underlying QTL. *β*_2 _is the log-transformed odds ratio for a user-specified one-unit increase of the continuous covariate. *G *× *E *is defined as the product of *G *and *E*, and *β*_3 _is the log-transformed odds ratio for this interaction term. *β*_0 _adjusts for the user-specified disease prevalence in the population of simulated individuals.

SIMLAPLOT evaluates QTL models, G × E interaction models, and genetic main effect models with covariate-defined heterogeneity. It produces four types of plots to explore different aspects of the relationship between affection status, continuous covariate values and marker genotypes in each model.

### Genotype-specific penetrance values as a function of covariate values

Three penetrance curves, one for each genotype, are produced. These curves display changes in penetrance as a function of *E*, if *E *is a risk factor for the simulated disease phenotype, either alone or in combination with genetic susceptibility.

### Conditional genotype probability as a function of covariate values and affection status

$$P(G|AFF,E)=\frac{P(AFF|G,E)*P(G,E)}{\sum_{G}P(AFF|G,E)*P(G,E)}$$

Three frequency curves, one for each genotype, are produced. At each point on the x-axis, the sum of the three frequencies is 1.0. The respective frequencies change as a function of *E *if the genotypes correspond to a QTL, if there is interaction with an environmental covariate, or if *E *is an indicator of genetic heterogeneity.

### Covariate distribution for each genotype in affected individuals

$$P(E|AFF,G)=\frac{P(AFF|G,E)*P(G,E)}{\sum_{E}P(AFF|G,E)*P(G,E)}$$

### Covariate distribution for each genotype in unaffected individuals

$$P(E|UNAFF,G)=\frac{P(UNAFF|G,E)*P(G,E)}{\sum_{E}P(UNAFF|G,E)*P(G,E)}$$

The covariate distributions are plotted for each genotype, separately for affected and unaffected individuals. The comparison of the two plots reflects the main effect of *E*, or the strength of G × E interaction.

SIMLAPLOT will plot the theoretical conditional distributions for the different models given the following input parameters: mean and standard deviation for *E*, which may or may not be genotype-dependent, allele frequency for the susceptibility locus, QTL or nearby marker, all relevant odds ratios, the mode of inheritance, and the type of model (model-based: QTL, G × E, or heterogeneity). Some parameters, such as genotype-specific means and variances, can be estimated from an existing data set, and some parameters are approximated based on the assumed model, e.g., QTL. SIMPLAPLOT also produces the same types of plots based on the observed data (data-based). Comparison of the observed to the theoretical distributions may suggest an appropriate model for the observed data set. To produce these plots SIMLAPLOT uses a kernel density estimate of the form $f(x)=\frac{1}{nb}\sum_{j=1}^{n}K(\frac{x-x_{j}}{b})$ with different kernels and width *b *[2]. Kernel options include Gaussian (the default), rectangular, triangular, and cosine. It is very important to evaluate the robustness of the visual plot appearance to the choice of smoothing parameters. SIMLAPLOT determines the optimal degree of smoothing by either minimizing the mean squared error (default) or minimizing the mean distance to the center-matched Gaussian predictions [5].

We applied SIMLAPLOT to the GAW15 simulated data sets using the quantitative covariates IgM, anti-CCP (anti-cyclic citrinullated protein), and severity of RA (rheumatoid arthritis). We analyzed all SNP markers on chromosomes 9, 11, and 18. Because covariate values exist only for affected individuals, we specified a relative risk of 1.0 and focused on two types of plots: the conditional genotype probability (plot type 2) and the covariate distribution for each genotype in affecteds (plot type 3). The input parameters for an assumed QTL model (plots labeled "model-based"), such as genotype-specific mean and variance, were estimated from the observed data for the specified SNP. We demonstrate SIMLAPLOT with data from Replicate 1. To evaluate our qualitative conclusions, we performed quantitative trait association analysis using the Monks-Kaplan method [3] as implemented in the QTDT program [4]. *p*-Values and their ranks were obtained for all 100 simulated replicates.

## Results

### Continuous covariate: IgM

**Figure 1:**
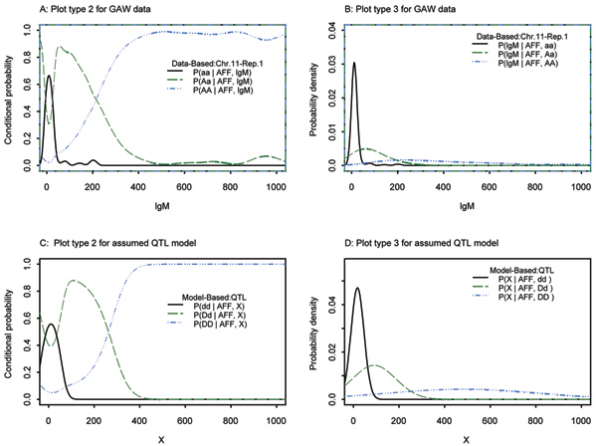
lgM, SNP389, chromosome 11.

**Figure 2:**
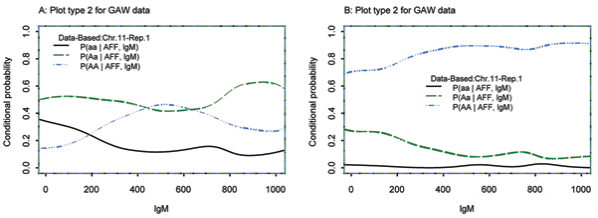
lgM, SNP387 (A), SNP388 (B), chromosome 11.

When SIMLAPLOT was applied to the continuous covariate IgM with markers on chromosome 11, three markers (SNPs 387, 388, and 389) were identified as potential QTL loci (Figs. 1 and 2). Figure 1A and 1B were based on the observed GAW15 data for Replicate 1 at SNP 389, the marker in highest LD with Locus F, which is a QTL for IgM. Figure 1A (plot type 2) demonstrates the strong dependence of genotype frequency on the IgM level. Figure 1B (plot type 3) shows that the genotypes define three different distributions. Figure 1C and 1D were produced by specifying a QTL model with the genotype-specific mean and variance estimated from the GAW15 data for SNP 389. To distinguish the theoretical distribution from the observed distribution, we use X to denote the covariate and dd, Dd, DD to denote the genotypes for the assumed QTL (SNP 389). The plots of the observed and theoretical distributions show remarkable agreement.

Figure 2A and 2B correspond to plot type 2 for SNPs 387 and 388, respectively. These plots reflect the weaker dependence of SNP genotype frequencies on covariate values with decreasing LD between alleles at the marker and QTL. Plots for markers even farther away demonstrate no dependence between the covariate and genotype (data not shown). Based on these plots, we conclude that SNP 389 is most significantly associated with IgM, and in highest LD with Locus F, the true QTL. These results were confirmed by the Monks-Kaplan analysis as implemented in QTDT (quantitative transmission-disequilibrium test). In Table 1 we summarize results from QTDT for all 100 replicates. SNP 389 was the most significant SNP associated with IgM. The *p*-value ranged from 10^-29 ^to 10^-42^. The next most significant SNPs were 387 and 388, with *p*-values lower than 10^-5^.

**Table 1:** QTDT-Monks Kaplan results

		Rank^a^			
			
Chromosome	SNP	1	2	3	Range of *p*-values over 100 replicates
11	389	100%	0%	0%	1.00 × 10^-42 ^to 9.00 × 10^-29^
	388	0%	10%	58%	5.00 × 10^-9 ^to 0.4048
	387	0%	87%	11%	4.00 × 10^-14 ^to 6.00 × 10^-4^
18	269	100%	0%	0%	2.00 × 10^-13 ^to 7.00 × 10^-4^
	270	0%	1%	1%	1.00 × 10^-3 ^to 0.99
9	186	51%	20%	6%	1.00 × 10^-9 ^to 0.3366

^a^Rank derived from ordering *p*-values for all SNPs on the same chromosome from lowest to highest.

### Continuous covariate: anti-CCP

**Figure 3:**
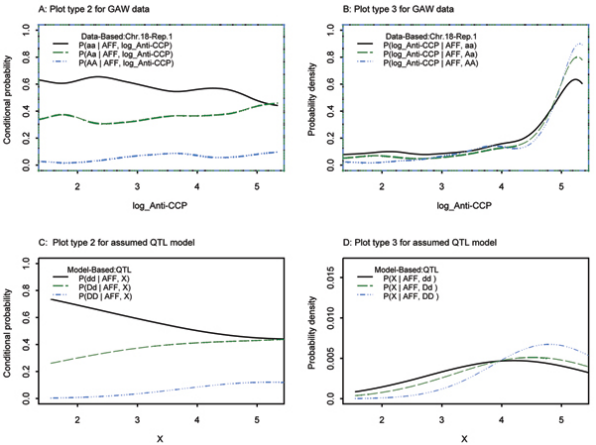
log Anti-CCP; SNP269, chromosome 18.

**Figure 4:**
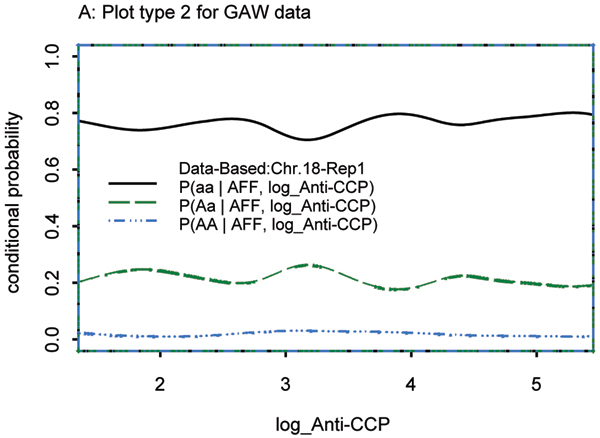
log Anti-CCP, SNP 270, chromosome 18.

When SIMLAPLOT was applied to anti-CCP with markers on chromosome 18, SNP 269 was identified as a QTL (Fig. 3). First, raw anti-CCP values were used but the plot type 3 from the theoretical distribution did not match the plot from the observed data very well (data not shown). When the log-transformation was applied to anti-CCP, both plot type 2 (Fig. 3A,C) and plot type 3 (Fig. 3B,D) provided a much better match in the region of interest. We note that plot type 2 based on the GAW15 data was fairly stable before and after applying the log-transformation to anti-CCP. Of interest is the plot of SNP 270, which is physically very close to SNP 269 but shows no association with anti-CCP values (Fig. 4). SNP 269 was also confirmed as a QTL by QTDT, since it yielded the lowest *p*-value of all SNPs on chromosome 18 for all replicates of the simulated data. SNP 270 was excluded as a QTL by QTDT, since the Monks-Kaplan test was not significant in 92% of the simulated replicates (Table 1).

### Discrete covariate: severity

**Figure 5:**
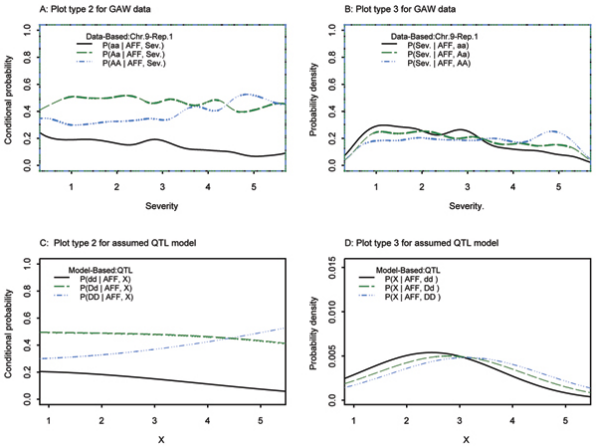
Severity, SNP186, chromosome 9.

SIMLAPLOT was designed to visualize a continuous covariate from a mixed normal distribution. In order to evaluate SIMLAPLOT using the severity trait, a discrete variable with only five values, we added a random uniformly distributed value between -0.5 and 0.5 to each severity value. As expected, the plot type 3 for SNP 186 in the GAW data did not match the plot type 3 from the QTL model very well (Fig. 5B,D), but we found that plot type 2 for the theoretical model and observed data for SNP 186 were quite similar to each other (Fig. 5A,C), both showing a dependence of genotype frequencies on severity. Thus, SIMLAPLOT was useful even in less than ideal situations. QTDT generated *p*-values as low as 10^-9 ^for SNP 186 in some replicates, and the *p*-value for this SNP ranked first for 51% of the GAW15 replicates (Table 1). Thus, both SIMLAPLOT and QTDT provided support for association of SNP 186 with the nearby QTLs for RA severity.

## Discussion

It is a challenge to identify the role of a continuous covariate in complex human diseases. We developed SIMLAPLOT as a visualization tool to explore different models by which continuous covariates may influence disease risk and to estimate parameters of interest. Our applications of SIMLAPLOT suggest that SNPs in strong LD with QTLs may be apparent when observed and expected (theoretical) plots of conditional genotype distributions across covariate values are compared. SIMLAPLOT may also help differentiate QTL models from interaction and heterogeneity models involving continuous covariates by comparing plots for affected and unaffected individuals.

## Competing interests

The author(s) declare that they have no competing interests.
